# Size Control of Synthesized Silver Nanoparticles by Simultaneous Chemical Reduction and Laser Fragmentation in *Origanum majorana* Extract: Antibacterial Application

**DOI:** 10.3390/ma14092326

**Published:** 2021-04-30

**Authors:** Entesar Ali Ganash, Reem Mohammad Altuwirqi

**Affiliations:** Physics Department, Faculty of Science, King Abdulaziz University, Jeddah 21589, Saudi Arabia; eganash@kau.edu.sa

**Keywords:** silver nanoparticles, *Origanum majorana* extract, pulsed laser fragmentation in liquids, size control, chemical reduction, antimicrobial activity

## Abstract

In this work, silver nanoparticles (Ag NPs) were synthesized using a chemical reduction approach and a pulsed laser fragmentation in liquid (PLFL) technique, simultaneously. A laser wavelength of 532 nm was focused on the as produced Ag NPs, suspended in an *Origanum majorana* extract solution, with the aim of controlling their size. The effect of liquid medium concentration and irradiation time on the properties of the fabricated NPs was studied. While the X-ray diffraction (XRD) pattern confirmed the existence of Ag NPs, the UV–Vis spectrophotometry showed a significant absorption peak at about 420 nm, which is attributed to the characteristic surface plasmon resonance (SPR) peak of the obtained Ag NPs. By increasing the irradiation time and the *Origanum majora* extract concentration, the SPR peak shifted toward a shorter wavelength. This shift indicates a reduction in the NPs’ size. The effect of PLFL on size reduction was clearly revealed from the transmission electron microscopy images. The PLFL technique, depending on experimental parameters, reduced the size of the obtained Ag NPs to less than 10 nm. The mean zeta potential of the fabricated Ag NPs was found to be greater than −30 mV, signifying their stability. The Ag NPs were also found to effectively inhibit bacterial activity. The PLFL technique has proved to be a powerful method for controlling the size of NPs when it is simultaneously associated with a chemical reduction process.

## 1. Introduction

Nanoscience has attracted much attention across numerous disciplines due to its promising future. Nanomaterials can be fabricated by several methods; each method has its advantages and disadvantages. However, the basis of all techniques can be categorized into one of two approaches: the top-down, or bottom-up. One example of the top-down approach is the pulsed laser ablation in liquid (PLAL) technique. Recently, researchers have shown increasing interest in using the PLAL method [1]. This is attributed to the simplicity of the method and to the fact that it is a fast, one-step, ecofriendly green method [2]. It is beneficial in producing the desired size or shape of nanostructures by changing either the laser parameters, such as energy, wavelength, pulse width, and fluence, or the ablated material parameters, including bulk, powders, and solution [3]. Furthermore, PLAL assists in producing nanostructures of high purity, dispersibility, and stability [4]. This has considerably opened up the field for the synthesis of various nanomaterials. This technique has been widely used to produce nanoparticles (NPs) of oxides [5], metals [6], and other materials [7].

While PLAL involves the ablation of bulk targets in a liquid medium, pulsed laser fragmentation in liquids (PLFL) starts with suspensions. The use of suspensions instead of bulk targets simplifies the experimental set-up and improves the fabricated product. PLFL has been used to reduce the particle size. For example, PLFL helped to reduce the size of Al nanoparticles in liquid isopropanol from over 100 nm down to 10 nm [8]. Furthermore, PLFL was also employed to reduce the size of colloidal copper(i) iodide nanoparticles suspended in water or ethyl acetate, and the mechanism of the process has been investigated [9]. From an application point of view, PLFL was employed to synthesize ZnO nanoparticles that were incorporated with graphene to produce a hybrid photodetector [10]. Therefore, PLFL is considered a powerful and simple technique for minimizing the size of NPs.

Noble metal NPs have received considerable attention owing to their unique chemical and physical properties, such as stability, conductivity, catalytic activity, and quantum size effect [6]. The size of metal NPs, such as gold, copper, and silver, is at the heart of our understanding of physical and chemical properties [11]. These metals are characterized by the surface plasmon resonance (SPR) in their absorption spectra [6]. Among metals, silver (Ag) is characterized by the highest SPR band intensity. Ag NPs have diverse applications, including renewable energy [12]; water treatment [13,14]; and biomedical [15,16], antibacterial [17,18,19,20,21], therapeutic [22,23,24], catalytic [25,26], and many others uses.

Ag NPs can be synthesized in several ways, such as the chemical reduction method [20,21,27] or PLAL method [6,11,28]. Many studies use both methods individually. For example, Ganash [20] used an *Origanum majorana* extract to produce Ag NPs in a bio-reduction process, where thymol is regarded as the main chemical compound in marjoram that is responsible for the reduction of Ag^+^ cations. It is worth noting that marjoram (*Origanum majorana)* is called bardaqush in Arabic. It is a type of plant belonging to the Lamiaceae family that grows in Asia, North Africa, and Europe. The PLAL method was also used for the synthesis of Ag NPs [29]. This work used the second harmonic wavelength of an Nd: YAG laser (*λ* = 532 nm), with a 100 kHz repetition rate and 10 ns pulse duration, to irradiate a silver plate in deionized and ice water. This method resulted in the production of Ag NPs with average sizes of about 31 nm and 16 nm when submerged in deionized and ice water, respectively. Other work used PLAL and investigated the influence of several parameters on the produced silver and gold NPs, including laser energy, ablation time, and the distance between the focal point and target [6]. The Ag target was immersed in deionized water and then ablated by a Q-switched Nd: YAG Laser (1064 nm) with an 8 ns pulse width and a 6 Hz repetition rate. Their results showed that the Ag NP size increased (from 11.5 to 23.3 nm average diameter) by increasing laser energy. Furthermore, the concentration of NPs increased with a longer ablation time or a considerable target distance from the focal point. More studies reported the fabrication of Ag NPs using double-distilled ultrapure water [30,31,32], ethanol [30,33,34], acetone [30,35], and polyvinylpyrrolidone (PVP) [36,37] as an ablation medium in the PLAL technique. It was found that a PVP solution was the best stabilizer [36].

Even though the chemical reduction and PLAL methods are not new, numerous procedures still need to be investigated, one of which is the effect of combining the two methods into one process. For example, simultaneous laser ablation and chemical reduction was employed to synthesize Ni/Pd NPs to decorate multiwall carbon nanotubes for effective enhancement of hydrogen storage [38]. The NPs produced in their work came from two paths: ablation of a metal target by laser irradiation, and NPs produced by a chemical reduction process.

In this work, a chemical reduction method and a PLFL technique are used simultaneously to reduce the size of Ag NPs. The Ag NPs are formed via a chemical reduction process through a green route. The novelty in this work appears in the ability to control the Ag NP sizes fabricated in *Origanum majorana* extract solution using both the bottom-up and top-down approaches at the same time. It should also be mentioned that, to the best of our knowledge, the *Origanum majorana* extract solution was used as a liquid medium in the PLFL method for the first time. The *Origanum majorana* extract functioned as both a reduction and capping agent. Two different concentrations of *Origanum majorana* extract solution were used at various irradiation times. Finally, the synthesized NPs were tested for antibacterial activity.

## 2. Materials and Methods

### 2.1. Sample Preparation

All glassware was washed with acetone and then with distilled water to ensure that no contamination was present. The experiment was divided into two parts: the chemical reduction process, and the laser irradiation. In the first part, samples were prepared for chemical reduction following the same procedures described by [20]. Two stocks were prepared, one concerning the *Origanum majorana* extract solution, and another relating to silver nitrate (AgNO_3_) solution. For the first stock, dried *Origanum majorana* leaves were bought from a local store, washed, and then left until dried. Once dry, 20 g of *Origanum majorana* leaves was weighted; then, 250 mL of distilled water was added. The mixture was boiled using a water bath for about 2 h. It was kept overnight in the refrigerator for stability and then filtered. For the second stock, a solution containing ≈0.17 g (10^−2^ M) of AgNO_3_ (Aldrich) in 100 mL distilled water was prepared. To investigate the effect of *Origanum majorana* extract solution concentration on the synthesized Ag NPs, two different volumes from the first stock were chosen. Volumes of 0.3 mL and 1.2 mL were taken from the *Origanum majorana* extract solution and each mixed separately with a constant volume of 2.5 mL of the second stock, the AgNO_3_ solution. Distilled water was then added in a 25 mL volumetric flask, making the concentration of the AgNO_3_ in the samples 10^−3^ M. Samples were then irradiated by the laser beam immediately. The same method was used to prepare numerous samples to study the effect of different irradiation times. The laser irradiation process is explained in Section 2.2. After laser irradiation, the Ag NP solution was filtered using a 0.20 μm filter. All samples were named *x* Ag *y*, where *x* is the concentration of *Origanum majorana* extract, and *y* is the irradiation time in minutes.

### 2.2. Experimental Set-Up

**Figure 1:**
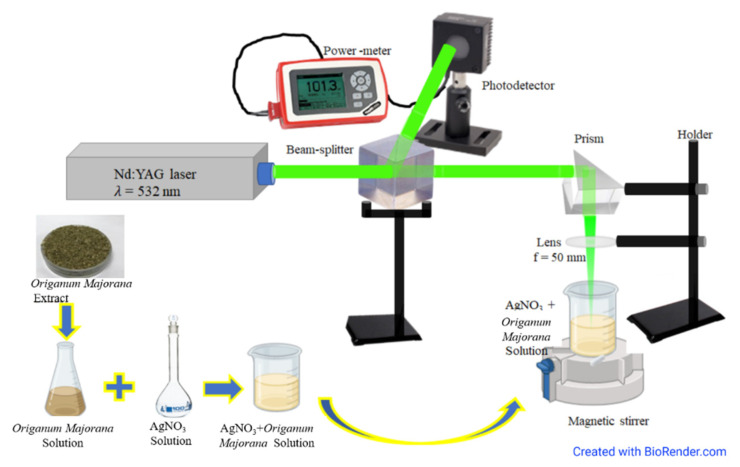
A schematic diagram of the experimental set-up.

A schematic illustration of the experimental set-up is shown in Figure 1. The second harmonic wavelength (532 nm) of a pulsed Q-switched Nd: YAG laser (Quanta Ray, Spectra-Physics) (Spectra-physics, Santa Clara, CA, USA) was focused on the sample using a lens (Thorlabs LB5284, CaF_2_ Bi-Convex Lens, f = 50.0 mm, uncoated) (Thorlabs, Newton, NJ, USA). The laser pulses had a repetition rate of 10 Hz with a duration of 6 ns. The laser output power was set to 0.1 W, which corresponds to a fluence of 0.02 J/cm^2^. To ensure stability, laser power was monitored throughout the experiment using a thermal power sensor (Surface Absorber, S350C, 0.19–1.1 μm and 10.6 μm, 40 W) (Thorlabs, Newton, NJ, USA) connected to a power meter (Thorlabs, PM 100A). As soon as the samples were prepared, as explained in Section 2.1, they were irradiated immediately in order to start the chemical reduction process and the laser irradiation simultaneously. Magnetic stirring of the sample was maintained during the irradiation process to allow homogeneous irradiation of the whole sample. The experiment was carried out at room temperature.

### 2.3. Characterization

Different characterization methods were used in this work. The optical properties were investigated using a UV–vis spectrophotometer (Thermo, Genesys 10 S model) (Thermo Fisher Scientific Inc., Waltham, MA, USA). X-ray diffraction (XRD) XTRA (Thermo Fisher Scientific Inc.), CuK_α_ radiation, λ = 1.54 Å, operating at 40 kV and 40 mA, scanning angles in the range 30–85°, and a scanning step of 0.1°) was applied to study the structure of the produced particles. In addition, a zetasizer (Malvern Instruments, Nano series, MPT-ZS model, Malvern, UK) was utilized to determine the size and distribution of the resulting NPs. In order to reveal the shape and size of the fabricated NPs, a transmission electron microscope (TEM) (JEOL, Peabody, MA, USA) (JEM-1400, V_max_ = 120 kV, resolution = 0.2 nm, and maximum magnification = 12 × 10^5^X) was employed.

## 3. Results and Discussion

**Figure 2:**
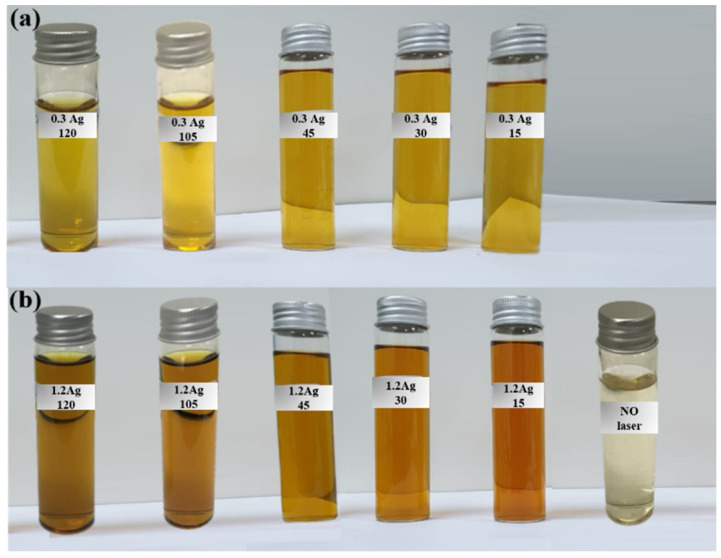
The prepared samples of two concentrations of *Origanum majorana* extract solution: (**a**) 0.3 mL and (**b**) 1.2 mL at different irradiation times.

The prepared samples of different *Origanum majorana* extract concentrations of 0.3 mL and 1.2 mL at different irradiation times (15, 30, 45, 105, and 120 min) are shown in Figure 2.

**Figure 3:**
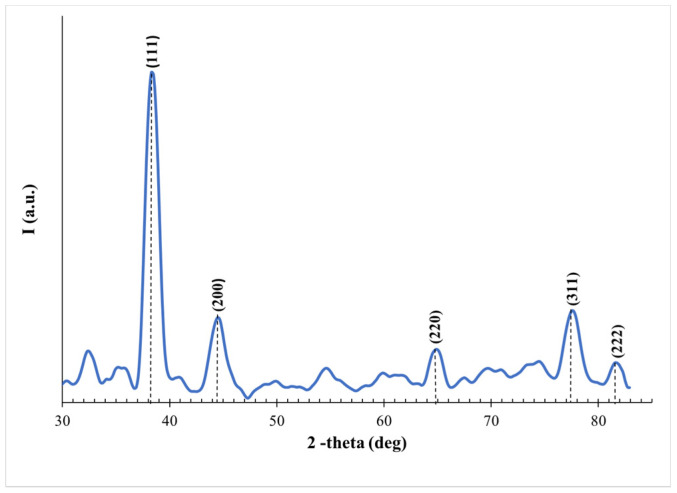
XRD spectrum of the produced Ag NPs.

The crystalline nature of the samples can be determined by investigating the XRD spectrum. To use this analysis, the solution of the sample was centrifuged (CAPPRondo Clinical Centrifuge, CRC-658) (CAPP, Nordhausen, Germany) at 6500 rpm for one hour, then washed with distilled water, and centrifuged again for one hour. The precipitated part was then deposited carefully on a glass substrate and left until dried. The XRD spectrum is demonstrated in Figure 3. The diffraction peaks indicate the presence of Ag with different lattice planes (111), (200), (220), (311), and (222) at 2θ = 38.3°, 44.5°, 64.9°, 77.5°, and 81.5°, respectively [18,39,40].

**Figure 4:**
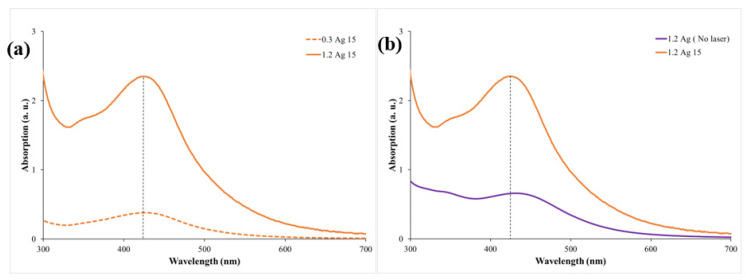
UV–vis absorption spectra of Ag NPs (**a**) using two concentrations of *Origanum majorana* extract with the same irradiation time, and (**b**) with and without laser irradiation.

**Figure 5:**
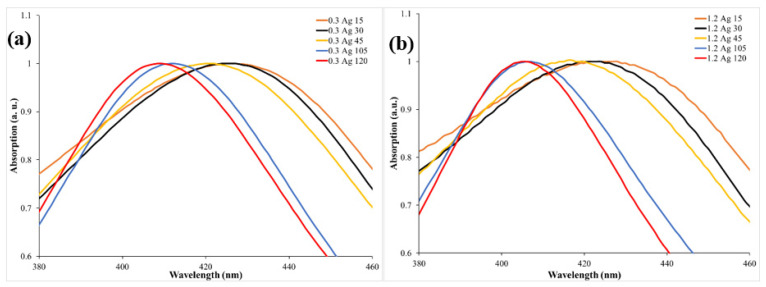
UV–vis absorption spectra of Ag NPs using two concentrations of *Origanum majorana* extract solution: (**a**) 0.3 mL and (**b**) 1.2 mL at different irradiation times.

Furthermore, the optical properties of the prepared samples were investigated. The absorption spectra of the samples for the two *Origanum majorana* extract concentrations, 0.3 mL and 1.2 mL, were collected using a UV–vis spectrophotometer in the wavelength range 190–1100 nm. To highlight the effect of *Origanum majorana* extract concentrations on the prepared samples, two samples that had different concentrations but were irradiated for the same period of time were chosen for comparison. The absorption spectra of samples 0.3Ag15 and 1.2Ag15 are shown in Figure 4a. Both samples’ spectra showed a peak at around 425 nm. The peak’s presence is attributed to the characteristic surface plasmon resonance (SPR) peak of fabricated Ag NPs. This peak is a consequence of the interaction between an incident resonance light on the NPs’ surface and the conduction electrons of Ag metal [12]. It can also be concluded from Figure 4a that an increase in the concentration of the *Origanum majorana* extract resulted in an increase in the absorption peak. This implies a rise in the number of fabricated Ag NPs. Figure 4b displays the effect of the chemical reduction and PLFL methods, when applied simultaneously, on the synthesis process in comparison to the sole effect of the chemical reduction. When the process of chemical reduction occurs individually, an SPR peak is seen at around 434 nm. However, when the PLFL method takes place simultaneously, the SPR peak is shifted to 425 nm. The peak shift towards a shorter wavelength indicates a change in the size and shape of the synthesized NPs, since the SPR peak depends on both [2]. By determining the absorption peak, the size of the synthesized NPs can be predicted [11,30]. Thus, the effect of the PLFL technique in reducing Ag NP size was significant, as suggested by the shift towards shorter wavelengths. Figure 4 shows the presence of only one SPR peak at around 400 nm, which is attributed to spherical-shaped Ag NPs. Triangular Ag NPs usually exhibit an SPR peak that is shifted toward a longer wavelength [41]. This peak was not apparent in our samples. Furthermore, to examine the effect of irradiation times on the size of the produced NPs, different irradiation times (15, 30, 45, 105, and 120 min) were applied to the samples for both concentrations (0.3 mL and 1.2 mL) of *Origanum majorana* extract. The absorption spectra obtained are illustrated in Figure 5a,b, and the results are listed in Table 1. It is worth noting that, with increasing irradiation time, a shift in the SPR peak toward a shorter wavelength is observed. For example, a shift toward a shorter wavelength of 16 nm occurred when the irradiation time increased from 15 min to 120 min. After 120 min of irradiation, the SPR peak was at 409 nm for the 0.3 mL concentration of *Origanum majorana* extract, and 405 nm for the 1.2 mL concentration. This shift in the SPR peak toward shorter wavelengths indicates a decrease in the Ag NPs’ size, reaching a size of about 10 nm [42]. That is, for every 15 min of irradiation, the size was reduced by 2 nm, approximately. The decrease in size is attributed to the increase in the interaction time between the laser and the sample under study, which allows the breakage of the NPs into smaller particles with increasing time. Hence, control of the size of the synthesized NPs can be achieved by altering the irradiation time. This result was also observed in previous work [5].

**Figure 6:**
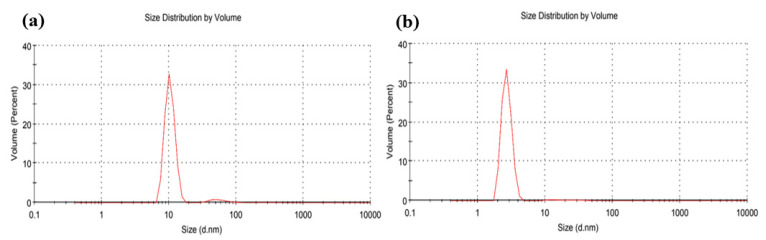
DLS size distribution of Ag NPs (**a**) without and (**b**) with simultaneous PLFL (120 min irradiation).

The dynamic light scattering technique (DLS) was used to determine the average sizes of the obtained Ag NPs. The DLS results show that the average diameter of the chemically reduced Ag NPs was about 10 nm when the process was without PLFL (Figure 6a). However, when PLFL was combined with the chemical reduction method, with 120 min of irradiation time, the average diameter of the synthesized Ag NPs was reduced to 3 nm (Figure 6b). Furthermore, the polydispersity index (PDI) is usually linked to the uniformity of the size distribution. In this work, it was found that the PDI was 0.47 when PLFL was not incorporated in the synthesis process and 0.32 when PLFL was combined. Since a smaller PDI indicates a more uniform particle distribution, including PLFL resulted in a more uniform distribution of the produced Ag NPs. To investigate the stability of the fabricated Ag NPs, the samples’ mean zeta potential was measured and found to be more than −30 mV, which indicates that the fabricated Ag NPs are generally stable. Hence, the use of the *Origanum majorana* extract as a liquid medium for the PLFL process functioned as a sufficient capping agent for the produced Ag NPs.

**Figure 7:**
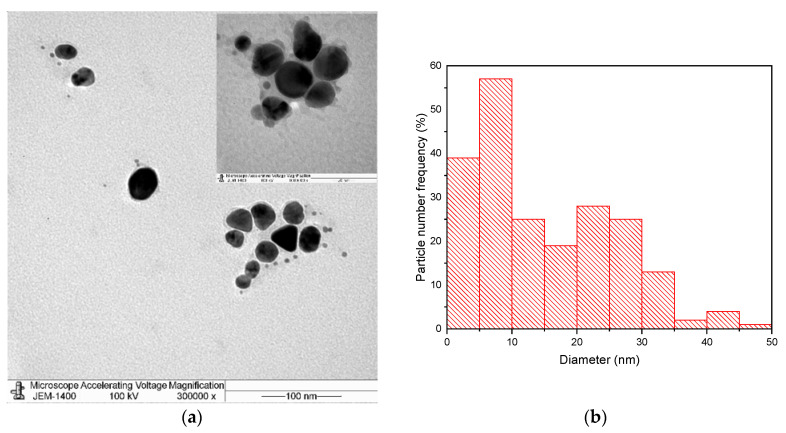

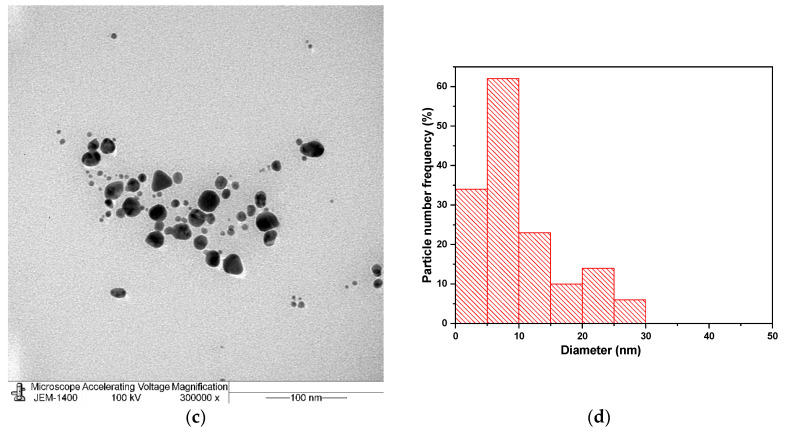
(**a**,**c**) TEM images of Ag NPs without and with laser irradiation, respectively. (**b**,**d**) The size distribution of these particles, correspondingly.

Figure 7a,b show a TEM image of Ag NPs produced by the chemical reduction process only and their size distribution, respectively. To study the effect of PLFL on size reduction, the TEM image and the corresponding size distribution of the sample when laser irradiation for 120 min was applied are illustrated in Figure 7c,d, respectively. It can be seen from the results that the size of Ag NPs without PLFL had a wide-spread range of diameters from 2 nm to 50 nm. However, when the PLFL process was incorporated, the size range of the NPs narrowed and varied from 2 nm to 27 nm, with the majority being less than 10 nm. Moreover, the size distribution became more uniform. This indicates that employing PLFL during the chemical reduction process had a significant role in the reduction in NP size, depending on experimental parameters.

The measurements obtained by DLS and TEM may differ, but this is expected as the two techniques are fundamentally different [43]. The difference in measurements becomes more significant when the sample is polydisperse. The TEM measurement is influenced by the number of particles studied which, in turn, affects the average diameter. On the other hand, DLS is based on the intensity of light that is scattered by the particles that constitute a larger sample size. By investigating Figure 6, it can be seen that there is a percentage of particles with larger diameters. These large particles affected the size distribution obtained by the TEM images (Figure 7b,d). Even though the measurement obtained from the two methods differ slightly, they both confirm that incorporating a PLFL process with a chemical reduction method did decrease the size of the fabricated NPs and narrowed the size distribution.

Regarding the morphology of the Ag NPs, the TEM images show spherical- and triangular-shaped Ag NPs. However, we believe that the concentration of triangular Ag NPs in our samples is minimal. Triangular Ag NPs usually exhibit an SPR peak that is shifted toward longer wavelengths [41]. The UV–vis spectra in this work (Figure 4) show only one SPR peak at around 400 nm, which is attributed to spherical-shaped Ag NPs and no other peak in the red region of the wavelength. Hence, it can be concluded that the formation of triangular Ag NPs is limited. The formation of triangular Ag NPs has been investigated extensively [41,44]. It was found that experimental parameters can affect the shape of the NPs. For example, changing the concentration of the metal ion and reducing agent can have an effect. In addition, the shape can also be controlled by selecting the irradiation wavelength. Since no significant concentration of triangular Ag NPs is present in our samples, we did not carry out a further investigation on the conditions affecting the formation of triangular Ag NPs.

### Ag NP Antimicrobial Activity

**Figure 8:**
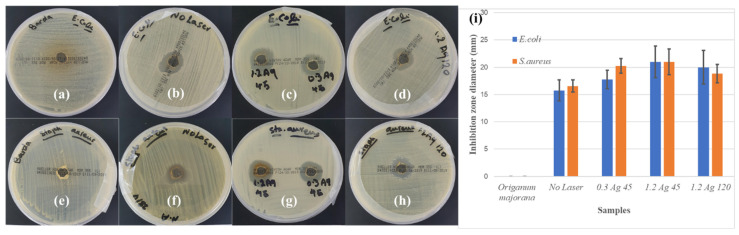
Inhibition zone photos of Ag NPs against microorganisms *E. coli* (top row) and *S. aureus* (bottom row) for (**a**,**e**) *Origanum majorana* extract solution, (**b**,**f**) chemical reduction only, (**c**,**d**,**g**,**h**) chemical reduction with simultaneous PLFL for different concentrations of *Origanum majorana* extract and different irradiation times, and (**i**) antimicrobial activity level of the produced Ag NPs.

The prepared Ag NP samples were used to test their effectiveness in inhibiting bacterial growth. In this work, Muller–Hinton agar (M.H agar) was used as an environment for microorganism growth. The prepared samples were tested against two types of bacteria: the Gram-negative bacteria *Escherichia coli* (*E. coli*), and the Gram-positive bacteria *Staphylococcus aureus* (*S. aureus*). About 30 μL of the different samples was added to these environments and then incubated for 24 h. The clear zones formed around the drops of the Ag NPs indicate the ability of the Ag NPs to inhibit the growth of the bacteria. The diameter measurements of the diffusion zone were carried out to evaluate the role of Ag NP samples as antimicrobic substances. The inhibition zone photos of Ag NPs against the microorganisms and the antimicrobial activity level of some of the produced Ag NPs are shown in Figure 8. It is clear from Figure 8a,e that the *Origanum majorana* extract on its own did not show any antimicrobial activity against both types of bacteria. However, the samples possessing Ag NPs showed an increased activity as deduced from the increase in the diameter of the inhibition zone (Figure 8i). Moreover, it can be seen from the comparison between Figure 8c,d,g,h and Figure 8b,f that the samples irradiated by the laser had a greater inhibition zone than those that were not irradiated. This could be attributed to the decrease in the size of Ag NPs in the laser irradiated samples resulting in a greater surface-to-volume ratio, allowing an increase in the interaction surface with the bacteria. Therefore, it can be concluded that the Ag NPs showed effectiveness in inhibiting the growth of the Gram-negative *Escherichia coli* (*E. coli*) and the Gram-positive *Staphylococcus aureus* (*S. aureus*) bacteria.

## 4. Conclusions

In this study, the chemical reduction method and the pulsed laser fragmentation technique were applied simultaneously in an *Origanum majorana* extract solution for the green synthesis of Ag NPs. The most important finding to emerge from the present work was the high efficiency of the PLFL technique for controlling the size of NPs when being associated with a chemical reduction method. Incorporating PLFL with a chemical reduction approach reduced the size of the Ag NPs to less than 10 nm. Furthermore, it was found that the reduction in NP size can be achieved by increasing the laser irradiation time. In fact, both the size and the size distribution were improved by integrating PLFL into the production process, and more uniform NPs were achieved. The reduction in size of the synthesized Ag NPs also enhanced their antibacterial activity. To conclude, laser beam parameters and irradiation time in the PLFL process can be used as tools to control the size of the green synthesized Ag NPs.

## Figures and Tables

**Table 1:** SPR position and irradiation time for two concentrations of *Origanum majorana* extract solution: 0.3 mL and 1.2 mL.

Item	0.3 mL					1.2 mL				
Sample	0.3Ag15	0.3Ag30	0.3Ag45	0.3Ag105	0.3Ag120	1.2Ag15	1.2Ag30	1.2Ag45	1.2Ag105	1.2Ag120
Irradiation time (min)	15	30	45	105	120	15	30	45	105	120
Peak position (nm)	425	424	420	412	409	421	419	414	406	405

## Data Availability

All data generated or analysed during this study are included in the article.

## References

[1] Fazio E., Gökce B., De Giacomo A., Meneghetti M., Compagnini G., Tommasini M., Waag F., Lucotti A., Zanchi C.G., Ossi P.M. (2020). Nanoparticles Engineering by Pulsed Laser Ablation in Liquids: Concepts and Applications. Nanomaterials.

[2] Rafique M., Rafique M.S., Kalsoom U., Afzal A., Butt S.H., Usman A. (2019). Laser Ablation Synthesis of Silver Nanoparticles in Water and Dependence on Laser Nature. Opt. Quantum Electron..

[3] Semaltianos N.G. (2010). Nanoparticles by Laser Ablation. Crit. Rev. Solid State Mater. Sci..

[4] Zhang D.S., Göekce B., Barcikowski S. (2017). Laser Synthesis and Processing of Colloids: Fundamentals and Applications. Chem. Rev..

[5] Altuwirqi R.M., Albakri A.S., Al-Jawhari H., Ganash E.A. (2020). Green Synthesis of Copper Oxide Nanoparticles by Pulsed Laser Ablation in Spinach Leaves Extract. Optik.

[6] Norsyuhada W., Shukri W.M., Bakhtiar H., Islam S., Bidin N. (2019). Synthesis and Characterization of Gold-Silver Nanoparticles in Deionized Water by Pulsed Laser Ablation (PLAL) Technique at Different Laser Parameter. Int. J. Nanosci..

[7] Ganash E.A., Al-Jabarti G.A., Altuwirqi R.M. (2019). The Synthesis of Carbon-Based Nanomaterials by Pulsed Laser Ablation in Water. Mater. Res. Express.

[8] Smirnov V.V., Zhilnikova M.I., Barmina E.V., Shafeev G.A., Kobtsev V.D., Kostritsa S.A., Pridvorova S.M. (2021). Laser Fragmentation of Aluminum Nanoparticles in Liquid Isopropanol. Chem. Phys. Lett..

[9] Schaumberg C.A., Wollgarten M., Rademann K. (2015). Fragmentation Mechanism of the Generation of Colloidal Copper(I) Iodide Nanoparticles by Pulsed Laser Irradiation in Liquids. Phys. Chem. Chem. Phys..

[10] Charipar K., Kim H., Piqué A., Charipar N. (2020). ZnO Nanoparticle/Graphene Hybrid Photodetectors via Laser Fragmentation in Liquid. Nanomaterials.

[11] Hajiesmaeilbaigi F., Mohammadalipour A., Sabbaghzadeh J., Hoseinkhani S., Fallah H.R. (2006). Preparation of Silver Nanoparticles by Laser Ablation and Fragmentation in Pure Water. Laser Phys. Lett..

[12] Alghoraibi I., Zein R., Edwards B. (2017). Silver Nanoparticles: Advances in Research and Applications is Approaching. Silver Nanoparticles: Advances in Research and Applications.

[13] Dankovich T.A., Gray D.G. (2011). Bactericidal Paper Impregnated with Silver Nanoparticles for Point-of-Use Water Treatment. Environ. Sci. Technol..

[14] Moustafa M.T. (2017). Removal of Pathogenic Bacteria from Wastewater Using Silver Nanoparticles Synthesized by Two Fungal Species. Water Sci..

[15] Chaloupka K., Malam Y., Seifalian A.M. (2010). Nanosilver as a New Generation of Nanoproduct in Biomedical Applications. Trends. Biotechnol..

[16] Burdușel A.C., Gherasim O., Grumezescu A.M., Mogoantă L., Ficai A., Andronescu E. (2018). Biomedical Applications of Silver Nanoparticles: An Up-to-Date Overview. Nanomaterials.

[17] Pandey J.K., Swarnkar R.K., Soumya K.K., Dwivedi P., Singh M.K., Sundaram S., Gopal R. (2014). Silver Nanoparticles Synthesized by Pulsed Laser Ablation: As a Potent Antibacterial Agent for Human Enteropathogenic Gram-Positive and Gram-Negative Bacterial Strains. Appl. Biochem. Biotechnol..

[18] Gao M.J., Sun L., Wang Z.Q., Zhao Y.B. (2013). Controlled Synthesis of Ag Nanoparticles with Different Morphologies and their Antibacterial Properties. Mater. Sci. Eng. C.

[19] Wang H., Jiang Y., Zhang Y., Zhang Z., Yang X., Ali M.A., Fox E.M., Gobius K.S., Man C. (2018). Silver Nanoparticles: A Novel Antibacterial Agent for Control of Cronobacter Sakazakii. J. Dairy Sci..

[20] Ganash A.A. (2019). Electrochemical Properties and Mechanistic Study of the Green Synthesis of Silver Nanoparticles Using Bardaqush Extract Solution. Mater. Res. Express.

[21] Ibrahim H.M.M. (2015). Green Synthesis and Characterization of Silver Nanoparticles using Banana Peel Extract and their Antimicrobial Activity against Representative Microorganisms. J. Radiat. Res. Appl. Sci..

[22] Zhang X.F., Liu Z.G., Shen W., Gurunathan S. (2016). Silver Nanoparticles: Synthesis, Characterization, Properties, Applications, and Therapeutic Approaches. Int. J. Mol. Sci..

[23] Chung I.M., Park I., Seung-Hyun K., Thiruvengadam M., Rajakumar G. (2016). Plant-Mediated Synthesis of Silver Nanoparticles: Their Characteristic Properties and Therapeutic Applications. Nanoscale Res. Lett..

[24] Wei L.Y., Lu J.R., Xu H.Z., Patel A., Chen Z.S., Chen G.F. (2015). Silver Nanoparticles: Synthesis, Properties, and Therapeutic Applications. Drug Discov. Today.

[25] Dong X.Y., Gao Z.W., Yang K.F., Zhang W.Q., Xu L.W. (2015). Nanosilver as a New Generation of Silver Catalysts in Organic Transformations for Efficient Synthesis of Fine Chemicals. Catal. Sci. Technol..

[26] Shenashen M.A., El-Safty S.A., Elshehy E.A. (2014). Synthesis, Morphological Control, and Properties of Silver Nanoparticles in Potential Applications. Part. Part. Syst. Charact..

[27] Hashemi S.F., Tasharrofi N., Saber M.M. (2020). Green Synthesis of Silver Nanoparticles Using Teucrium Polium Leaf Extract and Assessment of their Antitumor Effects Against MNK45 Human Gastric Cancer Cell Line. J. Mol. Struct..

[28] Tsuji T., Thang D.H., Okazaki Y., Nakanishi M., Tsuboi Y., Tsuji M. (2008). Preparation of Silver Nanoparticles by Laser Ablation in Polyvinylpyrrolidone Solutions. Appl. Surf. Sci..

[29] Hamad A.H. (2020). Nanosecond Laser Generation of Silver Nanoparticles in Ice Water. Chem. Phys. Lett..

[30] Moura C.G., Pereira R.S.F., Andritschky M., Lopes A.L.B., Grilo J.P.D.F., Nascimento R.M.D., Silva F.S. (2017). Effects of Laser Fluence and Liquid Media on Preparation of Small Ag Nanoparticles by Laser Ablation in Liquid. Opt. Laser Technol..

[31] Qayyum H., Ali R., Rehman Z.U., Ullah S., Shafique B., Dogar A.H., Shah A., Qayyum A. (2019). Synthesis of Silver and Gold Nanoparticles by Pulsed Laser Ablation for Nanoparticle Enhanced Laser-Induced Breakdown Spectroscopy. J. Laser Appl..

[32] Qayyum H., Ahmed W., Hussain S., Khan G.A., Rehman Z.U., Ullah S., Rahman T.U., Dogar A.H. (2020). Laser Synthesis of Surfactant-Free Silver Nanoparticles for Toxic Dyes Degradation and SERS Applications. Opt. Laser Technol..

[33] Nikov R.G., Nedyalkov N., Atanasov P.A., Karashanova D.B., Dreischuh T., Gateva S., Daskalova A., Serafetinide A. (2017). Characterization of Colloidal Silver Nanostructures Produced by Pulsed Laser Ablation in Different Liquids. Proceedings of the 19th International Conference and School on Quantum Electronics: Laser Physics and Applications, Sozopol, Bulgaria, 26–30 September 2016.

[34] Valverde-Alva M.A., García-Fernández T., Villagrán-Muniz M., Sánchez-Aké C., Castañeda-Guzmán R., Esparza-Alegría E., Sánchez-Valdés C.F., Llamazares J.L.S., Herrera C.E.M. (2015). Synthesis of Silver Nanoparticles by Laser Ablation in Ethanol: A Pulsed Photoacoustic Study. Appl. Surf. Sci..

[35] Solati E., Mashayekh M., Dorranian D. (2013). Effects of Laser Pulse Wavelength and Laser Fluence on the Characteristics of Silver Nanoparticle Generated by Laser Ablation. Appl. Phys. A..

[36] Syafiuddin A., Salmiati, Salim M.R., Beng Hong Kueh A., Hadibarata T., Nur H. (2017). A Review of Silver Nanoparticles: Research Trends, Global Consumption, Synthesis, Properties, and Future Challenges. J. Chin. Chem. Soc..

[37] Al-Azawi M.A., Bidin N., Ali A.K., Hassoon K.I., Abdullah M. (2015). Effect of Liquid Layer Thickness on the Ablation Efficiency and the Size-Control of Silver Colloids Prepared by Pulsed Laser Ablation. Mod. Appl. Sci..

[38] Mehrabi M., Parvin P., Reyhani A., Mortazavi S.Z. (2018). Hybrid Laser Ablation and Chemical Reduction to Synthesize Ni/Pd Nanoparticles Decorated Multi-Wall Carbon Nanotubes for Effective Enhancement of Hydrogen Storage. Int. J. Hydrog. Energy.

[39] Menazea A.A. (2020). Femtosecond Laser Ablation-Assisted Synthesis of Silver Nanoparticles in Organic and Inorganic Liquids Medium and their Antibacterial Efficiency. Radiat. Phys. Chem..

[40] Mostafa A.M., Menazea A.A. (2020). Polyvinyl Alcohol/Silver Nanoparticles Film Prepared via Pulsed Laser Ablation: An Eco-friendly Nano-Catalyst for 4-Nitrophenol Degradation. J. Mol. Struct..

[41] Etacheri V., Georgekutty R., Michael K.S., Suresh C.P. (2010). Single Step Morphology-Controlled Synthesis of Silver Nanoparticles. MRS Online Proc. Libr..

[42] Oldenburg S.J. (2021). Silver Nanoparticles: Properties and Applications. https://www.sigmaaldrich.com/technical-documents/articles/materials-science/nanomaterials/silver-nanoparticles.html.

[43] Kaasalainen M., Aseyev V., Von Haartman E., Karaman D.S., Makila E., Tenhu H., Rosenholm J., Salonen J. (2017). Size, Stability, and Porosity of Mesoporous Nanoparticles Characterized with Light Scattering. Nanoscale Res. Lett..

[44] Khodashenas B., Ghorbani H.R. (2019). Synthesis of Silver Nanoparticles with Different Shapes. Arab. J. Chem..

